# Differences in human skin volatiles between populations with high and low attraction to mosquitoes

**DOI:** 10.1186/s13071-025-06738-7

**Published:** 2025-05-21

**Authors:** Zhihua Fan, Teng Zhao, Zhenyu Gu, Heting Gao, Xinyu Zhou, Haotian Yu, Dan Xing, Hui Wang, Chunxiao Li

**Affiliations:** 1 School of Basic Medical Sciences, Anhui Medical University, Hefei, 230032 China; 2 State Key Laboratory of Pathogen and Biosecurity, Beijing, 100071 China

**Keywords:** VOCs, Mosquitoes, Odor, SBSE‒HRGC‒MS

## Abstract

**Background:**

The attractiveness of mosquitoes to humans varies among individuals, with human volatile organic compounds (VOCs) playing a pivotal role in the mosquitoes’ host-seeking behavior. Differences between human volatiles detected by GC-MS can effectively modulate mosquito host selection.

**Methods:**

Participants were enrolled and then assessed for mosquito attraction via an olfactometer. Their skin volatiles were collected with a stir bar as the sorptive extraction and were analyzed with high-resolution gas chromatography-mass spectrometry (SBSE-HRGC-MS). These data were then integrated with principal component analysis (PCA), volcano plot analysis, and partial least squares discriminant analysis (PLS-DA) to identify differential compounds between high and low mosquito attraction groups. Odorants with repellent properties were screened and evaluated using behavioral bioassays to assess their impact on the attractiveness of *Aedes aegypti*.

**Results:**

From the 30 volunteers, 24 participants (12/12 with high/low attractiveness to mosquitoes) were enrolled. In the group with high mosquito attraction, human skin compounds such as *N*,*N*-dibutyl formamide (10.8%), decanoic acid (9.2%), and decanal (5.9%) were detected with high components. Conversely, in the low mosquito attraction group, relatively high levels of indole (0.9%), fury hydroxymethyl ketone (2.2%), and 2-hydroxy-3-methyl-2-cyclopentenone (0.8%) were observed. The results of two pathway analyses indicated that most of these compounds are associated with fatty acid metabolism, respectively. Three compounds—2-hydroxy-3-methyl-2-cyclopentenone, furfuryl hydroxymethyl ketone, and 1,2-cyclopentanedione—were identified as prominent candidates, exhibiting significant repellent efficacy in behavioral bioassays.

**Conclusions:**

In this study, the impact of differences among VOCs emitted by human skin on the host-seeking behavior of *Ae. aegypti* was investigated, providing insights for the development of novel mosquito baits and repellents.

**Graphical Abstract:**

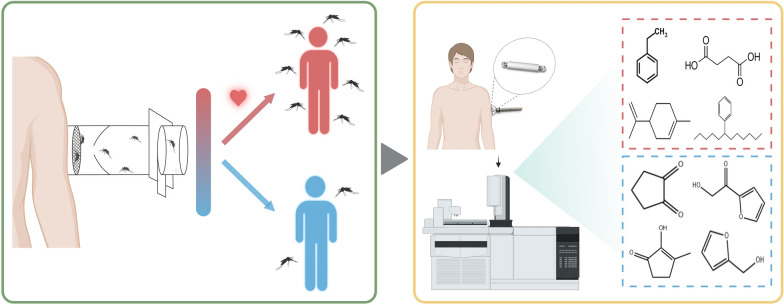

**Supplementary Information:**

The online version contains supplementary material available at 10.1186/s13071-025-06738-7.

## Background

Mosquitoes are “the world’s deadliest animal” [1]. In addition to causing itchy skin and nausea, mosquito bites are a primary cause of illnesses such as malaria [2], dengue fever [3], and Zika [4]. Mosquito-borne diseases significantly contribute to the global disease burden, posing a risk to the health and lives of nearly half the world’s population [5]. The most effective way to protect against these diseases is to reduce mosquito bites, and the use of insect repellents remains a key strategy. Therefore, a detailed investigation into the factors influencing mosquito attraction to humans is essential for developing more effective mosquito repellents.

The attraction of mosquitoes varies across different populations, with human odors and body temperature playing central roles in host selection. Certain individuals, such as pregnant women or those with infections, are more attractive to mosquitoes [6–9]. Carbon dioxide (CO₂) emitted by humans, along with clothing color [10–12], can stimulate mosquito behavior, attracting mosquitoes from a distance. In crowded settings, mosquitoes primarily rely on odor [13–16], body temperature [17], and other factors [18] to choose their hosts, with odor being the most influential [19], making it a central focus of research.

The odor of the human body arises mainly from volatile compounds produced by skin metabolism, including aldehydes, ketones, alcohols, carboxylic acids, hydrocarbons, and other substances [7, 20–22]. Differences in physiological traits and lifestyles lead to variations in these compounds between individuals, influencing mosquito host preferences. Identifying interindividual differences in metabolites could lead to the development of more targeted mosquito attractants or repellents, offering safer and more efficient alternatives to traditional plant-based and synthetic options. This research holds promises for future mosquito control products that minimize harm to both humans and the environment.

The application of sophisticated mass spectrometry has significantly enhanced the identification of skin surface compounds, particularly in the analysis of the chemical profiles of mosquito attractants, as reported in recent studies [23]. However, traditional sampling methods, such as fabric and sweat collection, have struggled to capture volatile odor molecules accurately. Passive sampling techniques, which use polydimethylsiloxane (PDMS) adsorbents in the form of bracelets or rollers, have emerged as effective tools for prolonged skin contact to collect skin volatiles [24]. Methods like stir bar sorptive extraction (SBSE), headspace solid-phase microextraction (HS-SPME), and dynamic headspace extraction (DHS) are valuable for capturing the volatile compounds that are difficult to sample using conventional methods [6, 7, 25–27].

This study utilized SBSE combined with high-resolution gas chromatography-mass spectrometry (HRGC-MS) to identify volatile compounds on the skin that may influence mosquito behavior. This method was highly effective in improving the detection rate of target compounds.

## Methods

### Volunteers

Thirty healthy volunteers (22 females and 8 males, aged 21–35) were recruited from the same unit. All volunteers followed similar lifestyle patterns. To control external factors, the participants were instructed to refrain from using perfumes, body lotions, hand creams, and other scented products. They were also advised to avoid alcohol and foods that could irritate the skin, such as raw garlic and onions, within 24 h prior to the experiment. Volunteers received financial compensation for their participation. Before beginning the experiment, they were informed of its details, potential discomforts, and recommended actions to mitigate any discomfort. All participants signed an informed consent form and provided accurate personal information.

### Mosquitoes

*Aedes aegypti* mosquitoes used in this study were collected from Mengpan Village in Xishuangbanna, Yunnan, China, and subsequently reared long term in the laboratory. The rearing environment was maintained at 26 ± 1 °C with 75 ± 5% humidity. Larvae were kept in enameled tubs with dechlorinated water and fed daily with fish food. Adult mosquitoes were housed in 25 cm × 25 cm × 30-cm mosquito cages and provided with an 8.0% sugar water solution.

### Mice

The female Kunming mice used in this study were supplied by Beijing Vital River Laboratory Animal Technology Co., Ltd. [license no. SCXK (Jing) 2016-0006], with body weights ranging from approximately 25 to 35 g. All animal experiments were approved and conducted under the guidance of the Institutional Animal Care and Use Committee (IACUC) of the State Key Laboratory of Pathogen and Biosecurity in compliance with ethical and welfare standards for laboratory animals.

### Mosquito attractiveness analysis

The experiment was conducted in a controlled environment with a temperature of 28 ± 1 °C and 60 ± 5% humidity. A polyethylene terephthalate (PET) device, designed to test mosquito behavior, was attached to the subject’s arm and positioned horizontally. Fifty female* Ae. aegypti* mosquitoes at 5–14 days post-eclosion (starved for 8–10 h) were introduced into the device. The volunteers wore medical protective masks and remained seated quietly at the experimental table to minimize physical activity during the test. Once the mosquitoes had acclimated, the baffle was removed, allowing the mosquitoes to choose freely for 1 h. Afterward, the number of mosquitoes in the capture area was counted. A blank control was set up in the same laboratory, away from the crowd, for comparison.

### Skin metabolite sampling with SBSE

Skin samples were collected immediately after the mosquito behavior test. A magnetic stirring bar (Twister) from Gerstel (Germany), equipped with a proprietary fixture for contact sampling, was used as the metabolite sampling device. The Twister surface was coated with polydimethylsiloxane (PDMS; 10 mm, 1 mm in film thickness; Gerstel), which is suitable for thermally resolved sampling [26, 28, 29]. Before use, the magnet was cleaned and aged in an external aging device (TC, Gerstel) at 260 °C for 60 min. The sampler was rotated 100 times at the sampling site, and after sampling, the magnetic beads were placed in a disposable injection vial with a sealed cap. The other Twister was exposed to air for 30 s before being placed into the vial as a control sample representing the sampling environment (blank). Disposable gloves were changed between samples from different subjects to prevent cross-contamination. The samples were stored at −20 °C, and GC–MS analysis was performed within 72 h.

### HRGC-MS analyses

#### Sample pretreatment

The Twister was rinsed with purified water at 35 °C, dried with dust-free paper to remove surface contaminants, and placed in an empty upper sample desorption tube.

#### Sample injection

The desorption tubes were then introduced into a thermal desorption system (TDU2, Gerstel) with an initial temperature of 30 °C, a ramp rate of 300 °C min, a desorption temperature of 240 °C, and a final hold time of 8 min. The system was operated in non-shunt mode. The cooled injection system (CIS) cold trap was set to an initial temperature of −40 °C, with a temperature increase rate of 10 °C/s, a desorption temperature of 240 °C, a final hold time of 8 min, and a 5:1 shunt ratio.

#### HRGC-MS

All the assays were performed via a mass analyzer equipped with a Gerstel MPS Robotic Pro autosampler, a thermal desorption system (TDU2), a Trace 1310 GC gas chromatograph, and a Q Exactive Orbitrap mass spectrometer (Thermo Fisher Scientific, Bremen, Germany). Helium (99.9999%) served as the carrier gas, with a constant flow rate of 1 ml/min. The column oven was programmed to begin at 40 °C for 2 min and then increased to 230 °C at a rate of 4 °C/min, where it was maintained for an additional 5 min. Mass spectrometry (MS) detection was conducted via electron ionization (EI) at 70 eV in full-scan mode, with a scanning range of 30–400 m^z−1^. The ion source and transfer line temperatures for MS were set at 280 and 250 °C, respectively. To mitigate the potential impact of ambient air on the samples, empty desorption tubes without a Twister were introduced during the detection process.

#### Compounds for mosquito repellency screening

In preliminary screening, compounds demonstrating differential bioactivity underwent efficacy evaluation to identify candidates with repellent properties. Experimental cohorts consisted of 100 female *Ae. aegypti* mosquitoes housed in 25 × 25 × 25-cm^3^ cages. Test compounds were dissolved in anhydrous ethanol at three concentrations (0.1, 0.01, and 0.001 mg/μl) under fume hood conditions. Prior to exposure, a 4-cm^2^ dorsal region was depilated on immobilized mice using a rodent restrainer. Following application of 200 μl anhydrous ethanol (5-min evaporation period), animals were secured in disposable latex gloves with only the treatment area exposed. Each prepared mouse was introduced into a mosquito cage for 2-min observation. Mosquito bites required ≥ 2 probing events within this interval; cohorts failing this threshold were excluded from subsequent trials. Post-validation, precisely measured compound aliquots (equivalent to ethanol control volume) were administered to depilated regions. Protection duration was assessed using the identical bite-test protocol established for ethanol controls, with fresh biological replicates (both mosquitoes and mice) employed for each compound to prevent cross-contamination. Biting capacity was re-verified before each experimental session.

#### Data analysis

The mosquito behavior test data were categorized into high and low groups based on attraction rates, with further analysis by sex and BMI. The SBSE-HRGC-MS data were acquired and processed using Xcalibur 4.1 and TraceFinder 4.0 software (Thermo Scientific) to facilitate the identification of volatile compounds based on the criteria outlined in the NIST17 and IAS Center Lab protocols. The compounds detected by SESE-GC-MS were analyzed by comparing and visualizing the main trends through principal component analysis (PCA), volcano analysis, and partial least squares discriminant analysis (PLS-DA), following log10 transformations and auto-scaling of the data via MetaboAnalyst 5.0. Differentially abundant volatile compounds were identified using a threshold of VIP > 1.0, distinguishing significantly different compounds from others. GraphPad Prism 9.0 and R were used to create charts.

## Results

### Variability in human attractiveness to mosquitoes

**Fig. 1:**
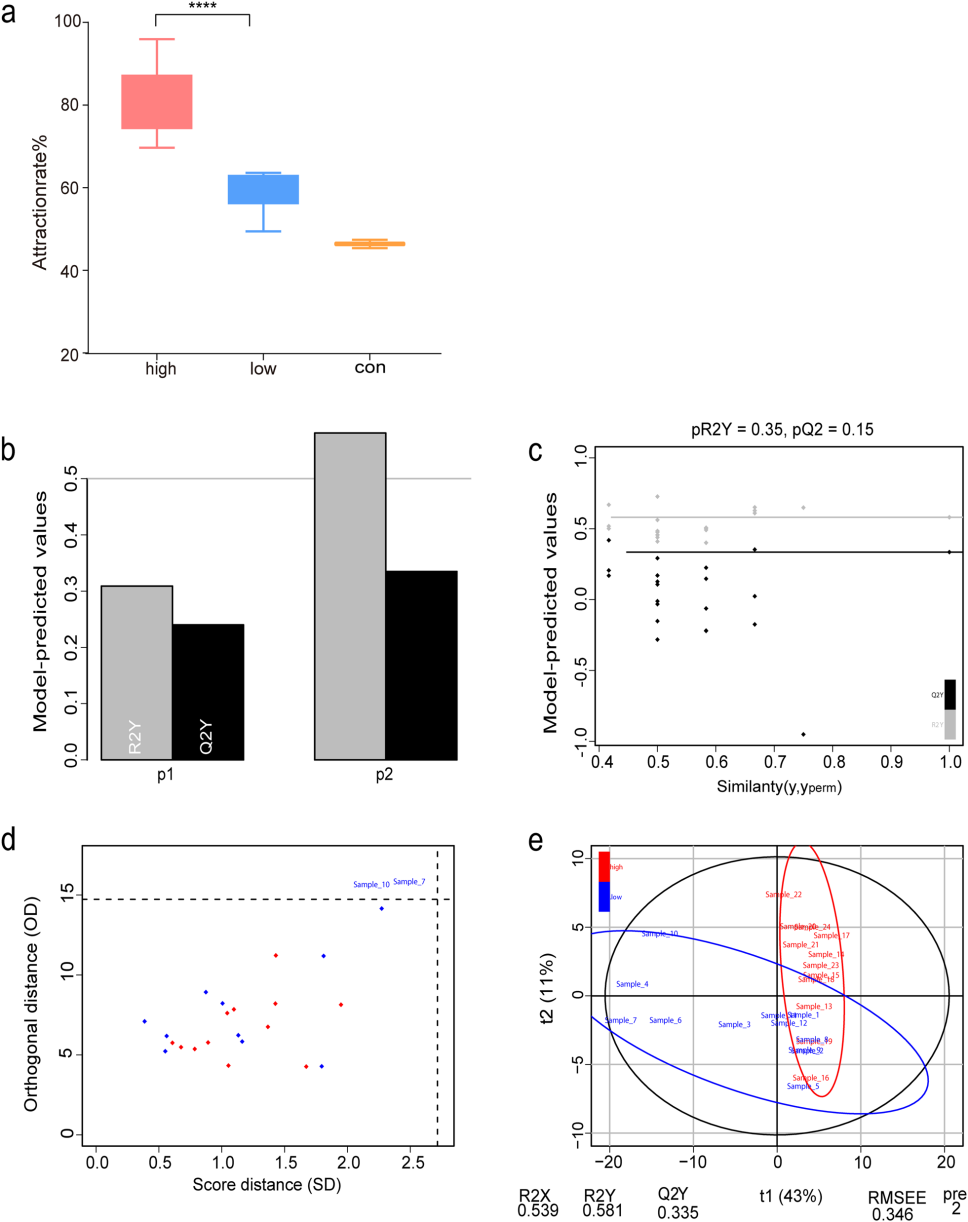
Response of *Aedes aegypti to volunteers.* **a** The subjects were classified into two groups based on their mosquito attraction rate: a high-attraction group and a low-attraction group. The t-test between the two groups yielded a *P*-value < 0.0001. **b–e** The predictive efficacy of the clustered models was evaluated based on several metrics: the variance contribution between the two groups, the sum of squared residuals, the orthogonal distance (OD), the score distance (SD), and the results from partial least squares discriminant analysis (PLS-DA)

A total of 30 participants were initially enrolled in this study, but six were excluded because of exposure to unusual conditions or skin disorders. The final analysis included 24 participants (male-to-female ratio of 1:3). Volunteers were categorized into three groups based on their BMI: obese (6), normal (11), and underweight (7). The majority of participants reported being particularly susceptible to mosquito bites, although 25% disagreed. To assess mosquito attraction, the participants were classified into two groups based on their attraction rate to *Ae. aegypti*, calculated as the number of mosquitoes attracted relative to the total number. The median attraction rate divided the participants into high (>70%) and low (<70%) attraction groups, with 12 individuals in each group. Both groups showed significantly higher attraction rates than the control group (Fig. 1a). Statistical analysis using a t-test (two-way) revealed significant differences in attraction rates across the groups (*P* < 0.0001) (Fig. 1b–e). Table 1 summarizes the mosquito behavior test results and questionnaire data, including gender, BMI, and self-reported mosquito attraction.

**Table 1:** Demographic information of subjects between high and low mosquito attraction groups

	Male	Female	BMI < 19	20 < BMI < 24	BMI > 24	Low subjective lure	High subjective lure
High attraction	3	9	3	7	2	3	9
Low attraction	3	9	1	8	3	3	9

Results from multiple logistic regression analysis showed no significant relationship between mosquito attraction and either sex or BMI, suggesting that the groups were comparable in terms of mosquito attraction ability.

### Compound analysis of mosquito-attractive human skin

**Fig. 2:**
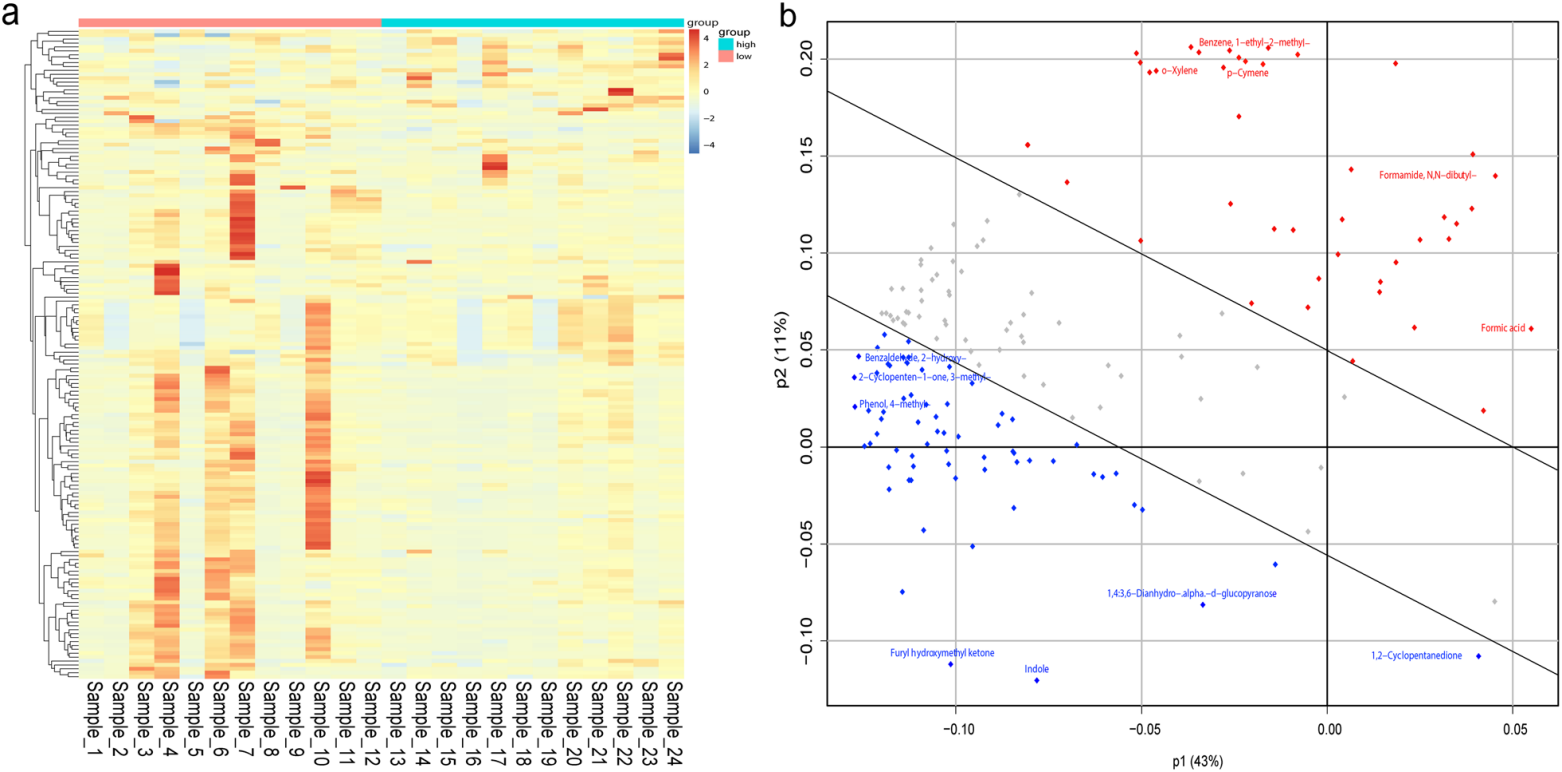
Composition of skin volatiles from different individuals. **a** A deeper red hue in the heatmap corresponds to higher compound concentrations, and the overall visual representation indicates that the high-attraction group exhibits higher compound concentrations compared to the low-attraction group. **b **The loading plot illustrates the degree to which individual metabolites contribute to the principal components, with the most significant contributors being 1,2-cyclopentanedione, 1,4:3,6-dianhydro-alpha-d-glucopyranose, furyl hydroxy methyl ketone, formic acid, *N*,*N*-dibutyl-formamide, and 1,4-diiodo-benzene. The gray dots represent compounds that contribute relatively less to the principal components

Using SBSE-HRGC-MS, we identified 698 volatile compounds from the skin surfaces of the participants (Table S1). The most common compounds included hydrocarbons, alcohols, aldehydes, ketones, esters, and heterocyclic compounds such as pyridines and pyrazines. After filtering data for delta retention index (RI) and removing duplicates, we identified 166 volatile molecules with a delta RI < 50 (Fig. 2a, Table S2). The primary components were identified as aldehydes (43), carboxylic acids and derivatives (37), ketones (28), and heterocyclic compounds (22). They contain only short- and medium-chain chemical molecules. For example, benzoic acid (7.8%), acetic acid (7.5%), 1-butanol (4.6%), benzaldehyde (4.4%), *N*,*N*-dibutyl-formamide (4.3%), decanoic acid (4.0%), heptyl ester-2-hydroxybenzoic acid (3.9%), phenol (3.6%), furfural (3.4%), decanal (2.5%), heptyl ester benzoic acid (2.4%), (*Z*)-6,10-dimethyl-5,9-undidecadien-2-one (2.0%), acetamide (1.9%), *n*-hexyl salicylate (1.8%), dimethyl phthalate (1.7%), and other compounds were the most prominent compounds (Fig. 2b). The least abundant compounds were methyl pyrazine, 4-octanone, and 2,2-dichlorocyclopropylbenzene. Some of these results may have been influenced by biological factors or environmental contamination.

### Compound difference between high and low mosquito-attractive human skin

**Fig. 3:**
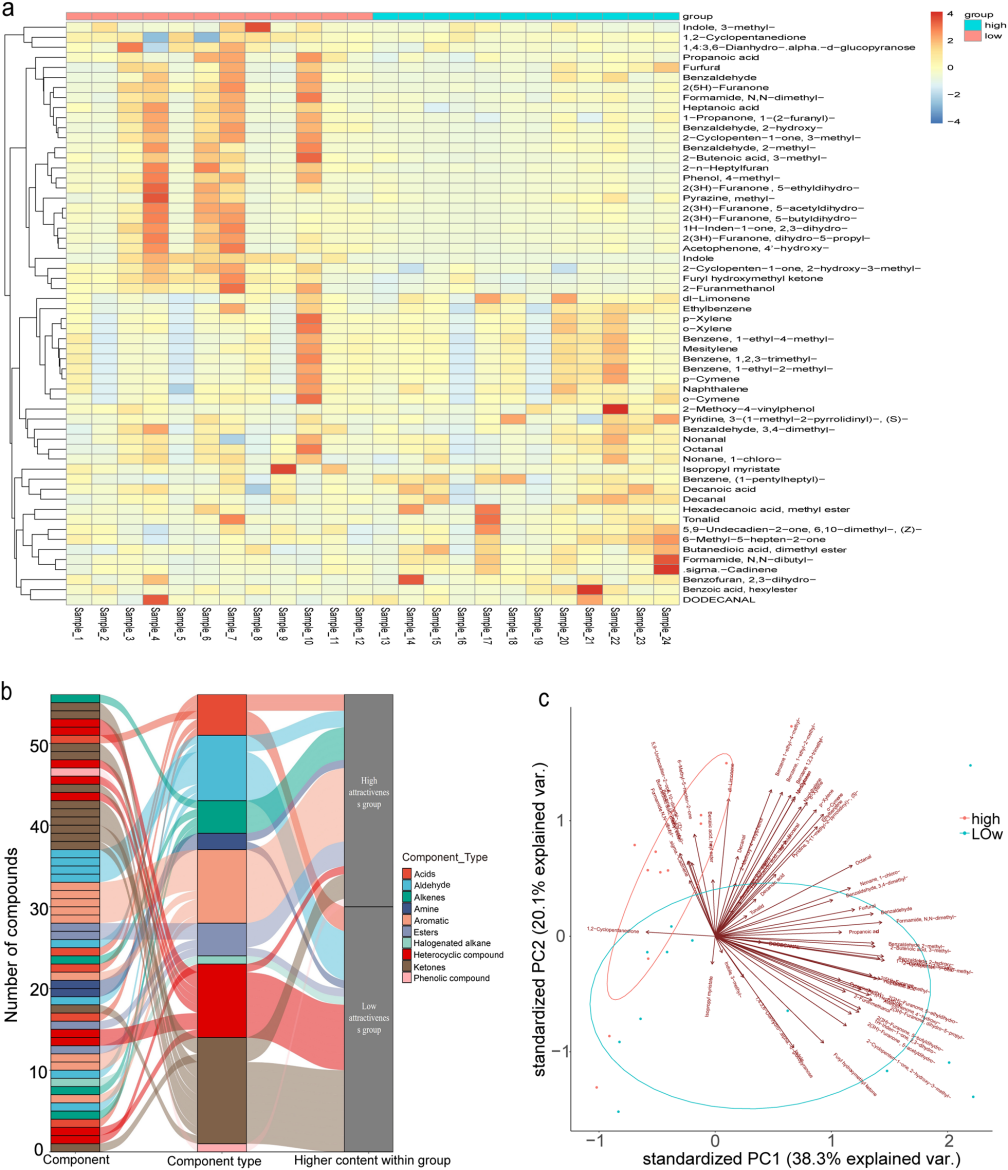
Differential compounds between groups with different mosquito attractiveness. **a** Heatmap quantifying the clustering of differential VOCs (VIP > 1.0) in the high- and low-attraction groups. **b** The Sankey diagram delineates the compound classes to which each differential VOC belongs and their corresponding groupings. Among the compounds, ketones and aromatics are notably prevalent. **c** The biplot illustrates the contribution of differential VOCs to the principal components within both groups

The differential chemicals were compared between the high- and low-attraction groups with a VIP > 1.0 criterion for differential volatile compounds. A total of 58 volatile compounds showed significant differences between the two groups (Fig. 3a), with hydrocarbons accounting for 20.7% of the total, carboxylic acids and their derivatives and ketones accounting for 19.0% each, and heterocyclic compounds and aldehydes accounting for 17.2 and 15.5%, respectively (Fig. 3b). The PCA results demonstrated that the biological replicates of the high-attraction group were distinctly separated from those of the low-attraction group by a 95% confidence ellipse and demonstrated the contribution of differential metabolites to the differences between the two groups (Fig. 3c).

In the low-attraction group, 31 compounds accounted for 53.5% of the differential compounds (Table 2). These compounds included indole (0.9%), fury hydroxymethyl ketone (2.2%), 2-hydroxy-3-methyl-2-cyclopentenone (0.9%), isopropyl myristate (5.2%), 2-furanmethanol (2.1%), 1,2-cyclopentanedione(3.9%),3,4-dimethyl-benzaldehyde(0.3%), and 1,4:3,6-dianhydro-α-d-glucopyranose. Most of these compounds were medium- (22) or short-chain (8) molecules, with isopropyl myristate being the only exception (C17).

**Table 2:** Differential compounds between high and low groups of mosquito attractiveness

Num	Group	Name	VIP value	CAS NO	Formula	Retention time (min)	Retention index
1	High	1-Pentylheptyl-benzene	1.9316	2719-62-2	C_18_H_30_	38.587	1919
2	High	*N*,*N*-Dibutyl-formamide	1.8469	761-65-9	C_9_H_19_NO	34.188	1758
3	High	Dimethyl ester butane dioic acid	1.6705	106-65-0	C_6_H_10_O_4_	28.804	1576
4	High	dl-Limonene	1.5917	138-86-3	C_10_H_16_	16.138	1193
5	High	Ethylbenzene	1.5647	100-41-4	C_8_H_10_	13.623	1105
6	High	Methyl ester hexadecenoic acid	1.4132	112-39-0	C_17_H_34_O_2_	45.409	2196
7	High	l-Nicotine	1.3924	1954/11/5	C_10_H_14_N_2_	50.463	1856
8	High	2,3-Dihydro-benzofuran	1.3705	496-16-2	C_8_H_8_O	49.125	2361
9	High	6-Methyl-5-hepten-2-one	1.3664	110-93-0	C_8_H_14_O	20.734	1335
10	High	(+)-δ-Cadinene	1.3346	483-76-1	C_15_H_24_	34.027	1752
11	High	o-Cymene	1.3146	527-84-4	C_10_H_14_	18.583	1271
12	High	o-Xylene	1.3128	95-47-6	C_8_H_10_	15.649	1175
13	High	Dodecanal	1.3081	112-54-9	C_12_H_24_O	32.517	1699
14	High	Decanal	1.2886	112-31-2	C_10_H_20_O	26.147	1493
15	High	p-Xylene	1.2720	106-42-3	C_8_H_10_	14.15	1123
16	High	Nonanal	1.2104	124-19-6	C_9_H_18_O	22.728	1391
17	High	Decanoic acid	1.2073	334-48-5	C_10_H_20_O_2_	46.387	2239
18	High	Hexyl ester benzoic acid	1.1739	6789-88-4	C_13_H_18_O_2_	43.662	2122
19	High	1-Ethyl-2-methyl-benzene	1.1499	611-14-3	C_9_H_12_	18.304	1262
20	High	1-Ethyl-4-methyl-benzene	1.1039	622-96-8	C_9_H_12_	17.042	1222
21	High	Naphthalene	1.0907	91-20-3	C_10_H_8_	33.596	1736
22	High	2-Methoxy-4-vinylphenol	1.0875	7786-61-0	C_9_H_10_O_2_	44.816	2171
23	High	*p*-Cymene	1.0657	99-87-6	C_10_H_14_	18.475	1268
24	High	(*Z*)-6,10-Dimethyl-5,9-undecadien-2-one	1.0653	3879-26-3	C_13_H_22_O	36.404	1837
25	High	Mesitylene	1.0410	108-67-8	C_9_H_12_	17.715	1244
26	High	1,2,3-Trimethyl-benzene	1.0253	526-73-8	C_9_H_12_	20.94	1340
27	High	Tonalid	1.0001	21145-77-7	C_18_H_26_O	49.54	2380
28	Low	Indole	2.2590	120-72-9	C_8_H_7_N	50.458	2420
29	Low	Fury hydroxymethyl ketone	1.9371	17678-19-2	C_6_H_6_O_3_	40.386	1988
30	Low	2-Hydroxy-3-methyl-2-cyclopentenone	1.6425	80-71-7	C_6_H_8_O_2_	35.555	1805
31	Low	Isopropyl myristate	1.4562	110-27-0	C_17_H_34_O_2_	41.131	2019
32	Low	2-Furanmethanol	1.4099	98-00-0	C_5_H_6_O_2_	30.685	1637
33	Low	1,2-Cyclopentanedione	1.4053	3008-40-0	C_5_H_6_O_2_	33.974	1750
34	Low	3,4-Dimethyl-benzaldehyde	1.3291	5973-71-7	C_9_H_10_O	35.569	1806
35	Low	1,4:3,6-Dianhydro-α-d-glucopyranose	1.3249	4451-30-3	C_6_H_8_O_4_	49.238	2367
36	Low	3-Methyl-indole	1.3114	83-34-1	C_9_H_9_N	51.553	2468
37	Low	Octanal	1.1844	124-13-0	C_8_H_16_O	19.202	1290
38	Low	2-*n*-Heptylfuran	1.1407	3777-71-7	C_11_H_18_O	24.011	1429
39	Low	Furfural	1.1382	1998/1/1	C_5_H_4_O_2_	24.885	1455
40	Low	Methyl-pyrazine	1.1005	109-08-0	C_5_H_6_N_2_	18.334	1263
41	Low	Heptanoic acid	1.0998	111-14-8	C_7_H_14_O_2_	38.735	1924
42	Low	Benzaldehyde	1.0859	100-52-7	C_7_H_6_O	26.966	1518
43	Low	5-Acetyloxolan-2-one	1.0840	29393-32-6	C_6_H_8_O_3_	41.066	2016
44	Low	2(5*H*)-Furanone	1.0781	497-23-4	C_4_H_4_O_2_	33.754	1742
45	Low	4-Hexanolide	1.0748	695-06-7	C_6_H_10_O_2_	33.606	1737
46	Low	4-Methyl-phenol	1.0724	106-44-5	C_7_H_8_O	40.069	1976
47	Low	Propanoic acid	1.0701	1979/9/4	C_3_H_6_O_2_	27.039	1520
48	Low	3-Methyl-2-cyclopenten-1-one	1.0634	2758-18-1	C_6_H_8_O	26.842	1514
49	Low	4-Heptanolide	1.0566	105-21-5	C_7_H_12_O_2_	35.192	1792
50	Low	4′-Hydroxy-acetophenone	1.0430	99-93-4	C_8_H_8_O_2_	35.201	1793
51	Low	2,3-Dihydro-1*H*-inden-1-one	1.0429	83-33-0	C_9_H_8_O	40.801	2005
52	Low	2-Hydroxy-benzaldehyde	1.0425	1990/2/8	C_7_H_6_O_2_	31.715	1672
53	Low	1-Chloro-nonane	1.0371	2473-01-0	C_9_H_19_Cl	21.402	1354
54	Low	1-(2-Furanyl)-1-propanone	1.0260	3194-15-8	C_7_H_8_O_2_	28.446	1565
55	Low	3-Methyl-2-butenoic acid	1.0167	541-47-9	C_5_H_8_O_2_	34.666	1774
56	Low	*N*,*N*-Dimethyl-formamide	1.0062	1968/12/2	C_3_H_7_NO	20.482	1328
57	Low	2-Methyl-benzaldehyde	1.0033	529-20-4	C_8_H_8_O	29.94	1612
58	Low	5-Butyldihydro-2(3*H*)-furanone	1.0026	104-50-7	C_8_H_14_O_2_	38.209	1904

In contrast, a total of 27 volatile compounds were considerably more common in the high-attraction group, comprising 49.0% of the total peak area for differentiated compounds (Table 2). These compounds were primarily medium- and long-chain molecules (C6–C18). The most abundant chemicals were *N*,*N*-dibutyl formamide (10.8%), decanoic acid (9.2%), decanal (5.9%), (*Z*)-6,10-dimethyl-5,9-undecadien-2-one (4.8%), and 6-methyl-5-hepten-2-one (3.1%).

### Compound pathway analysis

**Fig. 4:**
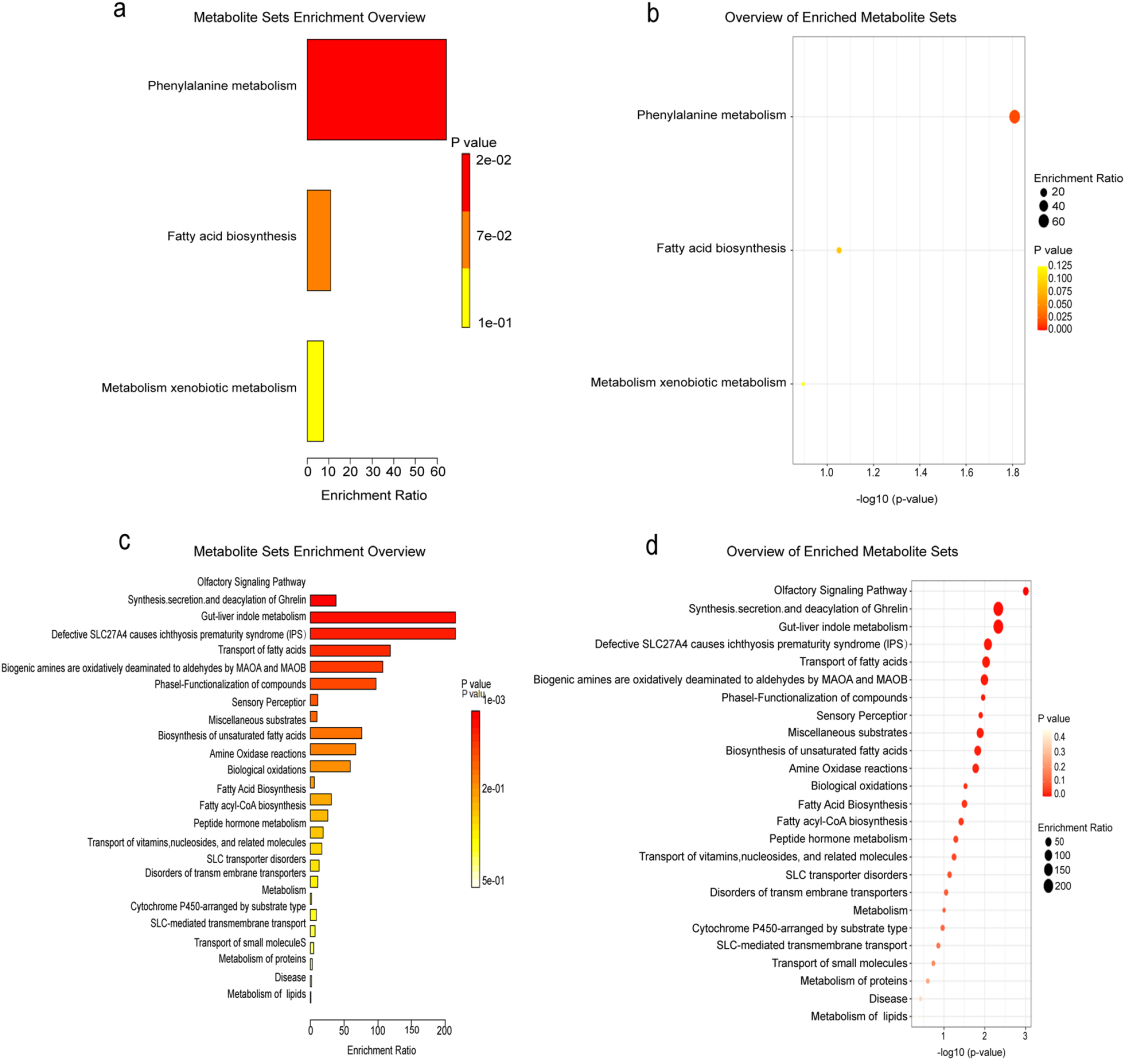
Pathways for differential volatile compound enrichment. **a**, **b** KEGG enriches differential VOCs in three pathways: phenylalanine metabolism, fatty acid biosynthesis, and xenobiotic metabolism via cytochrome P450. **c**, **d** RaMP-DB enrichment analysis revealed that 58 differentially expressed compounds were significantly enriched in a total of 25 pathways, predominantly those involved in fatty acid synthesis and transport, olfactory and perceptual signaling pathways, growth hormone-releasing peptide synthesis, secretion, and diacylation

The differential VOCs were enriched in KEGG (Kyoto Encyclopedia of Genes and Genomes) and RaMP-DB (Metabolomic Pathway Relationships Database) pathways to better understand the effects of odor molecules on mosquito behavior. The KEGG enrichment results revealed that differential VOCs were strongly enriched in three pathways: phenylalanine metabolism, fatty acid biosynthesis, and xenobiotic metabolism via cytochrome P450 (Fig. 4a, b). The RaMP-DB enrichment results revealed that 58 differentially expressed compounds were enriched in a total of 25 pathways, of which 14 pathway enrichment results were statistically significant, primarily fatty acid synthesis and transport (*P* < 0.05), olfactory and perceptual signaling pathways (*P* < 0.001), growth hormone-releasing peptide synthesis, secretion and diacylation, and biological oxidative reactions (Fig. 4c, d).

### Compound repellency analysis

**Fig. 5:**
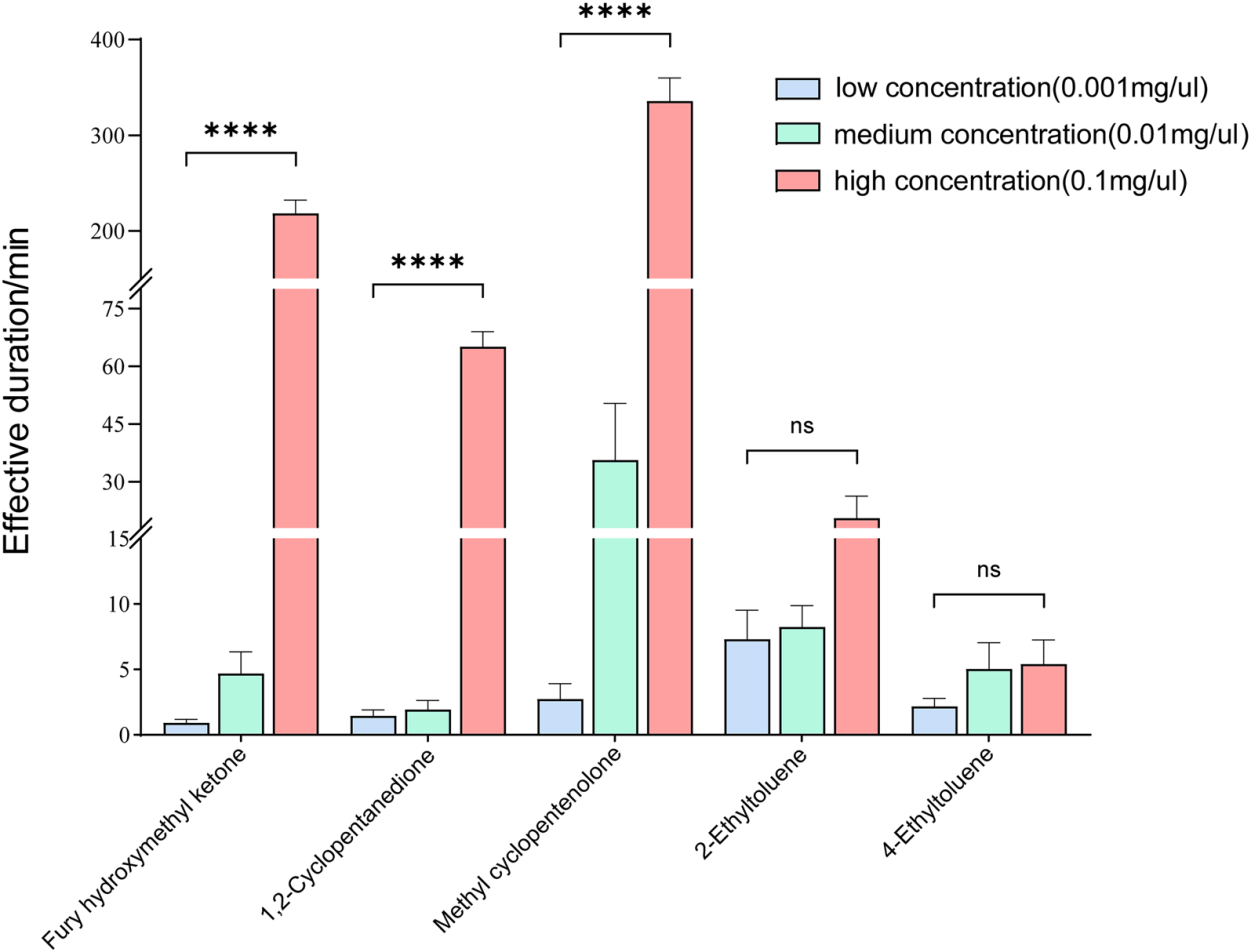
The repellent effect of odor molecules on mosquitoes. At elevated concentrations, furfuryl hydroxymethyl ketone, 1,2-cyclopentanedione, and 2-hydroxy-3-methyl-2-cyclopentenone demonstrated significant mosquito repellent efficacy, whereas neither low nor medium concentrations exhibited substantial repellent activity (*P* < 0.0001). Across all three tested concentrations, both 2-ethyltoluene and 4-ethyltoluene failed to demonstrate satisfactory levels of mosquito repellent activity

This investigation systematically evaluated five candidate compounds for their repellent efficacy against female *Ae. aegypti* under controlled laboratory conditions. Dose-response analysis (Fig. 5) revealed concentration-dependent efficacy profiles among the test compounds. At the high concentration (0.1 mg/μl), furfuryl hydroxymethyl ketone demonstrated sustained protection with a mean effective duration of 218.4 ± 19.8 min (> 3 h), while 1,2-cyclopentanedione provided 64.8 ± 7.8 min of complete protection. Notably, 2-hydroxy-3-methyl-2-cyclopentenone exhibited exceptional repellency, maintaining mosquito bite prevention for 336.0 ± 48.6 min (> 6 h). In contrast, both low (0.001 mg/μl) and intermediate (0.01 mg/μl) concentrations demonstrated suboptimal repellent efficacy against mosquitoes under standardized testing protocols. Additionally, this study also tested two compounds highly expressed in highly attractive individuals: 2-ethyltoluene and 4-ethyltoluene. Under the same experimental conditions, neither compound demonstrated effective mosquito-repellent properties.

## Discussion

Previous studies have provided strong evidence that individuals exhibit varying levels of attractiveness to mosquitoes [30–32]. However, the specific process underlying this variability remains unknow. One widely accepted explanation attributes this variability to differences in the skin microbiota, which produces odor molecules that influence mosquito behavior [33, 34]. These skin odors were primarily metabolites from bacteria residing on the skin’s surface, and they were influenced by factors such as age, sex, genetics, occupation, and environmental conditions [35]. In our study, we focused on individuals from a homogeneous demographic, controlling environmental and physiological factors. This allowed us to investigate how differences in cutaneous volatile organic compounds (VOCs) contribute to variations in mosquito attraction. Using the Twister sampler for VOC collection was found to be effective in isolating skin metabolites while minimizing the impact of sweat and other external factors. This tool enabled us to identify differential compounds between individuals with high and low mosquito attractions, providing insight into the role of specific chemicals in mosquito behavior.

In this study, the presence of indoles in the low-attractant group was previously identified as a factor influencing mosquito behavior. Indoles and 3-methylindole are tryptophan degradation products [36]; when different compounds are combined, a luring or repellent effect has been observed on *Ae. aegypti* and *Culex quinquefasciatus* mosquitoes [37–42]. By inhibiting the activation of the mosquito olfactory receptor OR8, indoles significantly decrease the appeal of *Ae. aegypti* to human hosts [43]. This could be because it lessens the attractiveness of the attractant mixture made from molecules found in the skin [24, 44]. Furthermore, the cyclopentanone analog in this group is a human odor analog that produces robust and consistent stimulation of the mosquito’s CO_2_ receptor [45]. Additionally, research suggests that cyclopentanone, either by itself at high concentrations or in conjunction with substances such as lactic acid and ketones, has a strong effect on mosquitoes [45–47]. However, 1,2-cyclopentanedione exhibited significant repellent efficacy in our experimental system, providing complete protection against *Ae. aegypti* attacks in mice throughout the 6-h monitoring period. According to a review of the literature, the attraction effect of CO₂ on nearby *Ae. aegypti* mosquitoes was effectively reduced by a final concentration of 3.0% 2,3-butanedione, hexanol, butanal, and pentanal [48]. A previous study examining interindividual differences in mosquito attractiveness focused on different classes of compounds and revealed that 6-methyl-5-hepten-2-one, octanal, nonanal, decanal, and geranyl acetone were enriched in the skin of poorly attracted individuals [34]. This implies that certain people might emit natural repellents that reduce mosquito attraction. In this study, two additional ketone compounds screened—furfuryl hydroxymethyl ketone and 2-hydroxy-3-methyl-2-cyclopentenone—were also found at higher concentrations in individuals with lower attractiveness to mosquitoes, and both exhibited significant mosquito-repellent effects. The current line of mosquito repellents is hazardous to both people and the environment to varying degrees. Additionally, mosquitoes have become resistant as a result of their prolonged use. It is crucial to investigate these human-derived chemicals to create safer and more effective insect repellents.

The differential volatile compounds found in people with high levels of mosquito attraction may have an attractive effect on mosquitoes, which was also supported by other studies. For example, the widely reported compound 6-methyl-5-hepten-2-one, known as sulcatone [49, 50], is present at significantly higher levels in human skin than in other animals and can stimulate mosquito olfactory neurons, thereby inducing mosquito biting behavior [51], with an effect analogous to that of CO_2_ [46]. Logan’s team discovered that a mixture of compounds, including decanal, octanal, and nonanal, can effectively repel *Ae. aegypti* mosquitoes, with the repellent effect lasting for several hours [34, 52]. Furthermore, common skin-derived aldehydes have been widely studied for their potential role in the host-seeking behavior of anthropophilic blood-feeding mosquitoes. For example, decanal attracted *Aedes mcintoshi* and *Ae. ochraceus*, which are vectors of Rift Valley fever virus (RVFV) [53]. Both laboratory and field tests have been shown that (*E*)-6,10-dimethy-5,9-undecadien-2-one combined with substances such as benzaldehyde, 1-octen-3-ol, and p-cresol captures more Culex mosquitoes [54]. Electroantennography (EAG) experiments have demonstrated that decanoic acid at doses of 0.1, 1, and 10 µl can elicit a response in sedentary *Cx. quinquefasciatus* [55]. Similarly, Jane's research showed that a combination of lactic acid and ketoglutaric acid attracts *Ae. aegypti* mosquitoes [19]. Long-chain carboxylic acids (C10–C20), especially undecanoic, pentadecanoic, and heptadecanoic acids, were shown to be more prevalent in the dermal metabolites of mosquito-attractive persons according to a recent study on carboxylic acids impacting *Ae. aegypti* [7].

In this investigation, the KEGG database revealed high enrichment of differential chemicals in three pathways: fatty acid biosynthesis, phenylalanine metabolism, and cytochrome P450-mediated xenobiotic metabolism. This may be because these compounds were directly produced from substrates for fatty acid synthesis or metabolites of phenylalanine. An essential ingredient that mosquitoes acquire after feeding on blood is phenylalanine, an aromatic amino acid that is involved in a number of metabolic processes in living organisms [56, 57]. Fatty acids are important energy sources and structural components in living organisms [58], and the fatty acid biosynthetic pathway involves several enzymes and regulatory factors that work together to coordinate the fatty acid synthesis process [59]. When differential volatile compounds are significantly enriched in the fatty acid biosynthetic pathway, these compounds may be associated with fatty acid synthesis or regulation. It was hypothesized that mosquitoes can sense the location of the host via phenylalanine metabolites or substrates in the fatty acid synthesis process, resulting in differences in mosquito attraction ability.

The differential compounds identified in this study were enriched in the RaMP-DB database for fatty acid synthesis and transport, olfactory and perceptual signaling pathways, and synthesis growth factor effects on both body temperature and CO_2_ release [60, 61]. The perception of temperature and CO_2_ by mosquitoes has been reported in the literature [62–65]. The differentially abundant metabolites were also associated with olfactory and perceptual signaling pathways, which may exercise mosquito olfactory receptor function and regulate olfactory signaling [66, 67]. The VOC screening conducted in this study supports potential applications in developing biogenic mosquito repellents and contributes to current understanding of related pathways, including hormone-releasing peptides, secretion and diacylation, and biological oxidation processes. These processes generally encompass substance synthesis and metabolism, often accompanied by energy conversion during synthesis and catabolism, as well as energy release, transfer, storage, and utilization. Energy metabolism may play a significant role in mosquito infection dynamics.

Despite the encouraging results, several challenges remain in this field. Individual variations among volunteers may lead to the screening of a broader range of repellent compounds, complicating subsequent behavioral bioassaying. Future studies should involve a larger cohort of volunteers and collect more skin surface volatile samples to narrow the range of compounds screened for repellent efficacy. Alternatively, developing new repellent formulations by testing the effects of mixtures of screened compounds could be explored.

## Supplementary Information

Additional file 1: Table S1. The 698 volatile compounds were identified from the skin surfaces of the participants.

Additional file 2: Table S2. The 166 volatile molecules were screened with a delta RI < 50.

## Data Availability

No datasets were generated or analysed during the current study.
